# Pegylated liposomal doxorubicin (Lipo-Dox®) combined with cyclophosphamide and 5-fluorouracil is effective and safe as salvage chemotherapy in taxane-treated metastatic breast cancer: an open-label, multi-center, non-comparative phase II study

**DOI:** 10.1186/s12885-015-1433-4

**Published:** 2015-05-21

**Authors:** Kun-Ming Rau, Yung-Chang Lin, Yen-Yang Chen, Jen-Shi Chen, Kuan-Der Lee, Cheng-Hsu Wang, Hsien-Kun Chang

**Affiliations:** 1 Division of Hematology-Oncology, Department of Internal Medicine, Kaohsiung Chang Gung Memorial Hospital, Kaohsiung, Taiwan; 2 College of Medicine, Chang Gung University, Tao-Yuan, Taiwan; 3 Division of Hematology-Oncology, Department of Internal Medicine, LinKo Chang Gung Memorial Hospital, 5, Fushing St., Gueishan Township, Taoyuan, 333 Taiwan; 4 Division of Hematology-Oncology, Department of Internal Medicine, Chang Gung Memorial Hospital at Chiayia, Chiayia, Taiwan; 5 Graduate Institute of Clinical Medical Sciences, College of Medicine, Chang Gung University, Taoyuan, Taiwan; 6 Division of Hematology-Oncology, Department of Internal Medicine, Chang Gung Memorial Hospital at Keelong, Keelong, Taiwan

**Keywords:** Advanced breast cancer, Pegylated liposomal doxorubicin, Metastatic breast cancer, Taxane failure, Safety

## Abstract

**Background:**

Anthracycline and taxane are classes of drugs that are frequently used in the adjuvant and palliative settings of metastatic breast cancer (MBC); however, treatment failure occurs in most cases. Limited data demonstrated favorable response in MBC after previous taxane-based treatment. The aim of this study was to evaluate the efficacy and safety of pegylated liposomal doxorubicin (Lipo-Dox®) used as part of a combination salvage therapy for patients with MBC whose tumors progressed during or after taxane-based treatment.

**Methods:**

Patients with MBC who failed to respond to previous taxane-based treatments were recruited. Treatment with pegylated liposomal doxorubicin (40 mg/m^2^), cyclophosphamide (500 mg/m^2^), and 5-fluorouracil (500 mg/m^2^) was administered every 3 weeks. Tumor response to treatment was determined by using the Response Evaluation Criteria in Solid Tumor criteria version 1.0, and left ventricular ejection fraction was measured before and after treatment using echocardiography. Each patient was followed for 30 days after the last dose of study medication or until resolution/stabilization of any drug-related adverse event.

**Results:**

Forty-five patients were recruited. As of December 2012, the median follow-up duration was 29.8 months, the overall response rate was 41.9 %, the median progression-free survival was 8.2 months, and the median overall survival was 36.6 months for all treated patients. Grade 3/4 neutropenia, leucopenia, and neutropenic fever were observed in 14 %, 9 %, and 1 % of the cycles, respectively. Other non-hematologic adverse effects were mild to moderate and were manageable. No decrease in left ventricular ejection function was noted.

**Conclusion:**

This regimen of combined of pegylated liposomal doxorubicin, cyclophosphamide, and 5-fluorouracil exhibited a promising overall response rate, progression-free survival rate, and overall survival rate, with a safe cardiac toxicity profile and manageable adverse effects. This regimen could be considered as a treatment option for patients with MBC whose tumors progressed during or after taxane-based treatment.

## Background

Breast cancer is now the most frequently diagnosed cancer among women in 140 of 184 countries and the most common cause of cancer death among women (522,000 deaths in 2012), especially in less developed countries. Since 2008, the incidence and mortality rate of breast cancer has increased by more than 20 % and 14 %, respectively [1]. While the incidence of breast cancer remains highest in more developed regions, the mortality rate is much higher in less developed countries, primarily because early detection and access to treatment facilities are lacking. Although improvements in early detection and systemic therapy have significantly decreased recurrence and prolonged survival, metastatic breast cancer (MBC) is still a predominantly incurable disease [2–4]. With prolonged survival and tumor recurrence, serious problems emerge, including accumulated drug dosages that approach the upper limit of safety, therapy-related toxicity, and drug resistance. Consequently, there is an ever-increasing need for new drugs or combination regimens for the treatment of MBC.

Pegylated liposomal doxorubicin (PLD, Lipo-Dox®) is a formulation of doxorubicin in poly(ethylene glycol)-coated (stealth) liposomes. This formulation causes fewer cardiac events, has a longer half-life, and exhibits higher tumor tissue penetration compared to standard doxorubicin [5]. O’Brien et al. reported that, compared to doxorubicin, PLD provides equivalent progression-free survival (PFS; 7.8 vs. 6.9 months, respectively) and overall survival (OS; 22 and 21 months, respectively) when used as the first-line therapy for MBC [6]. As a maintenance therapy, the adverse effects of PLD are manageable and include bone marrow suppression, mucositis, and hand-foot skin reaction [6, 7].

Taxanes and/or anthracyclines are widely used as the initial therapy for breast cancer, as well as for adjuvant and palliative chemotherapy. Data are limited regarding effective treatment strategies for MBC that has recurred or progressed following taxane- and/or anthracycline-based treatment. A triweekly PLD-cyclophosphamide regimen has been reported to be effective and well tolerated as the first-line therapy for patients with metastatic or recurrent breast cancer [8, 9]. The aim of this study was to evaluate the efficacy and safety of a PLD-combined regimen as second-line treatment for patients with progressed MBC who had undergone a previous taxane-based treatment.

## Methods

This study was an open-label, multicenter, non-comparative prospective phase II clinical trial performed from August 2005 to July 2010 following approval by the Institutional Review Board Committee at the Chang Gung Memorial Hospital, Taiwan.

### Patient selection

Eligible patients included women with histologically proven MBC, presenting with at least one disease lesion measuring ≥ 20 mm in at least one dimension by conventional techniques or ≥ 10 mm by spiral computed tomography (CT) or magnetic resonance imaging (MRI). Enrolled patients were ≥20 years old with an Eastern Cooperative Oncology Group (ECOG) performance status ≤ 2 and had received a prior taxane-based chemotherapy regimen for metastatic disease. Biological criteria that were to be met before the first cycle of treatment were as follows: hemoglobin ≥ 10 g/dl, absolute neutrophil count (ANC) ≥ 1,500/μl, platelets ≥ 100,000/μl, total bilirubin ≤ 3.0 mg/dl, aspartate aminotransferase/alanine aminotransferase ≤ 2 × upper normal value, and creatinine ≤ 1.5 mg/dl. All patients received both oral and written information regarding the trial and provided written informed consent.

Exclusion criteria consisted of 1) a life expectancy of less than 3 months, 2) prior use of free anthracycline or PLD for MBC, 3) contraindication to anthracycline, fluorouracil (5-FU), or cyclophosphamide, 4) bone metastasis, 5) brain metastasis, 6) other malignancy except curative, treated non-melanoma skin cancer or cervical carcinoma *in situ*, 7) serious concomitant illness potentially aggravated by the study medication, including uncontrolled infection or active cardiac disease, 8) pregnancy or breast feeding, and 9) child-bearing potential unless a reliable contraceptive method is used throughout the treatment period and for 3 months following cessation of treatment.

### Trial design and treatment

Although the typical chemotherapeutic regimen involves a sequence of monochemotherapy, we used a combination of three therapies to obtain a synergistic effect. All eligible subjects received cyclophosphamide (500 mg/m^2^) and 5-FU (500 mg/m^2^) intravenous infusion (IVF) over 1 h, followed by Lipo-Dox® (40 mg/m^2^) IVF over 1 h on day 1 of each 21-day cycle. Dose modifications were permitted for hematologic and non-hematologic toxicity. Complete blood counts were checked on days 1 and 8. If the absolute neutrophil count was lower than 500/mm^3^, administration of granulocyte-stimulating factors was allowed. Treatment continued until progression, unacceptable toxicity, or the patient’s decision to withdraw from the study.

### Assessment

Tumors were assessed within the 21 days preceding chemotherapy and after every 3 cycles of chemotherapy. Tumor response was determined by using the Response Evaluation Criteria in Solid Tumors version 1.0. Each patient was followed for 30 days after the last dose of study medication or until resolution/stabilization of any drug-related adverse event.

### Statistical considerations

The primary endpoint was the overall response rate (ORR) of patients with MBC treated with Lipo-Dox® combined with cyclophosphamide/5-FU as a salvage treatment. The secondary endpoints included 1) PFS, defined as the time interval between the start date of treatment and the date of disease progression, death by any cause without progression, or the last follow-up without progression, 2) duration of response (DR), defined as the time interval between the onset of a clinical response and objective evidence of progression, death by any cause without progression, or last follow-up, and 3) OS, defined as the time interval between the start date of treatment and the date of death by any cause or last follow-up without death and the safety profiles.

At the end of the study, patients were categorized into evaluable and/or intent-to-treat (ITT) patient populations according to their termination status. The ITT population was defined as all patients exposed to at least one study regimen. The evaluable population was the subset of ITT patients who completed the baseline evaluation, who had at least one post-treatment evaluation, and who were exposed to at least three cycles of treatment.

Simon’s optimal two-stage design was used to determine the target patient number for this study. Drug treatment was considered inactive if the response probabilities were less than 20 %, while treatment was considered effective if response probabilities were greater than 40 %. ORR was assessed in both the evaluable and ITT population data sets; however, the main analysis was focused on the evaluable population. Efficiency was calculated as the number of responding patients divided by the number of all patients treated (i.e. ITT and/or evaluable patients). Descriptive statistics were used for the primary analysis, presented by a point estimate and 95 % confidence interval (CI) for the primary efficacy variable (ORR). The PFS, DR, and OS were evaluated using the Kaplan-Meier method.

### Safety

All safety analyses were performed on the safety population, which was defined as all the ITT patients available for a follow-up evaluation of safety. Incidences of adverse events were tabulated by severity and relationship to the treatment. Treatment toxicity was evaluated according to the National Cancer Institute Common Terminology Criteria for Adverse Events, version 4.0.

## Results

### Patient characteristics

From August 2005 to July 2010, a total of 45 women with MBC whose disease had progressed after prior treatment with a taxane-containing regimen were enrolled in the current study. The median age at the time of enrollment was 52.5 years. As of December 2012, the median follow-up period was 29.8 months. Twenty percent of the patients had metastasis to one organ, 24.4 % had metastasis to two organs, and 55.6 % had metastasis to more than two organs. All patients had failed the previous taxane-based treatment for MBC and only seven patients had previously received an anthracycline-based regimen as adjuvant therapy. The majority of patients had an ECOG score of 1 (80.0 %). Twenty-six patients (58 %) had an estrogen receptor (ER)-positive tumor, and adjuvant hormonal therapy was administered for cases with indications. This trial pre-dated the routine use of trastuzumab for MBC in Taiwan; therefore, six patients did have the Her2 status of their tumor tested (Table 1). Two patients, who had received one cycle of treatment each, withdrew informed consent and dropped out of the trial. The median number of chemotherapy cycles received by the ITT and evaluable groups was 5.7 and 5.9, respectively (Table 2).

**Table 1:** Patients’ baseline characteristics

	Number (%)
Median Age, years	52.5
ECOG performance status	
0	4 (8.9)
1	36 (80.0)
2	5 (11.1)
Initial stage at diagnosis	
I	6 (14.6)
II	20 (38.8)
III	6 (14.7)
IV	9 (22.0)
Metastatic site	
Locally advanced	4 (8.9)
Regional lymph nodes	7 (15.6)
Distant lymph nodes	14 (31.1)
Lung	18 (40.0)
Liver	17 (37.8)
Bone	25 (55.6)
Skin/Soft tissue	8 (17.8)
Others	13 (28.9)
Number of metastatic sites	
0-1	9 (20.00)
2	11 (24.44)
≥3	25 (55.56)
Previous anthracycline	
Yes	7 (16.28)
No	36 (83.72)
Estrogen receptor	
Positive	26 (57.78)
Negative	16 (35.56)
Unknown	3 (6.67)
Her-2 expression^a^	
0, 1+	30 (66.67)
2+	4 (8.89)
3+	5 (11.11)
Unknown	6 (13.33)

Abbreviations: ECOG, Eastern Cooperative Oncology Group

^a^Her-2 expression was determined by immunohistochemical staining

**Table 2:** Treatment exposure

Treatment cycle	ITT* (*n* = 45)	Evaluable (*N* = 43)
Mean (SD)	5.7 (3.0)	5.9 (2.9)
Median (min-max)	5.0 (1.0-12.0)	5.0 (1.0-12.0)

*ITT: intent-to-treat

### Efficacy

**Fig. 1:**
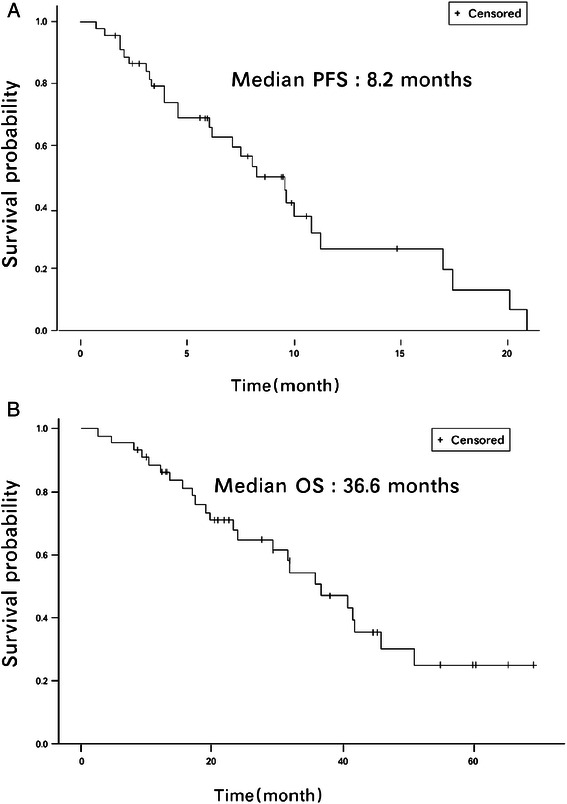
**(a)** Progression free survival (PFS) and **(b)** Overall survival (OS) of intent-to-treat (ITT) patients. The median PFS was 8.2 months, and the median OS was 36.6 months

Efficacy analyses were based on the total patients enrolled (i.e. the evaluable and ITT populations). Because only two patients were not evaluable in the ITT group, the efficacy evaluation was essentially the same for these two groups. In the ITT and evaluable populations, 36 patients achieved stable disease (SD) or partial response (PR) as their best response. Disease control rates (DCR) were nearly identical in the ER-positive (PR of 46 %, DCR of 88.5 %) and Her2-positive populations (PR of 20 %, DCR of 80.0 %) (Table 3). We also checked the response rate at different metastatic sites, lymph nodes had the best response. In general, most visceral organs had response rates more than 50 % (Table 4). The PFS and OS of the ITT patients were identical to those of the evaluable patients (Fig. 1; median PFS = 8.2 months, median OS = 36.6 months). For patients who achieved partial response, the median PFS was 9.96 months and the median OS was 41.48 months, as compared to the patients who achieved SD, who had a median PFS of 6.16 months and a median OS of 36.62 months (Table 5).

**Table 3:** Treatment response in different populations

	ITT population (*N* = 45)	Evaluable population (*N* = 43)	Estrogen receptor positive (*N* = 26)	Her-2 positive (*N* = 5)
Tumor response				
CR, n (%)	0 (0.0 %)	0 (0.0 %)	0 (0.0 %)	0 (0.0 %)
PR, n (%)	18 (40.0 %)	18 (41.9 %)	12 (46.2 %)	1 (20.0 %)
SD, n (%)	18 (40.0 %)	18 (41.9 %)	11 (42.3 %)	3 (60.0 %)
PD, n (%)	7 (15.6 %)	7 (16.3 %)	2 (7.7 %)	1 (20.0 %)
NE, n (%)	2 (4.4 %)	0 (0.0 %)	1 (3.8 %)	0 (0.0 %)
Disease control rate				
CR + PR + SD, n (%)	36 (80.0 %)	36 (83.7 %)	23 (88.5 %)	4 (80 %)
Objective response rate				
CR + PR, n (%)	18 (40.0 %)	18 (41.9 %)	12 (46.2 %)	1 (20.0 %)

Abbreviations: CR, complete response; PD, progressive disease; PR, partial response; SD, stable disease; NE: not evaluable

**Table 4:** Response rate evaluated by site of metastasis

	Number of responsive lesions	Number of evaluable lesions	Response rate (%)
Liver	26	39	66.7
Lung	10	18	55.6
Lymph node	39	54	72.2
Skin	5	8	62.5
Others	9	20	45.0

**Table 5:** Progression free survival and overall survival

	ITT population (*N* = 45)	Evaluable population (*N* = 43)	PR Population (*N* = 45)	SD Population (*N* = 45)
Median PFS (95 % CI)	8.2 mo (6–10.8)	8.2 mo (6–10.8)	9.96 mo (8.03-17.38)	6.16 mo (3.9-16.95)
Median OS (95 % CI)	36.6 mo (23.8-45.8)	36.6 mo (23.8-45.8)	41.48 mo (23.21-NA)	36.62 mo (17.51-NA)

Abbreviations: CI, confidence interval; ITT, intent-to treat; mo, months; NA, not available; PFS, progression-free survival; PR, partial response; SD, stable disease

### Safety

Most adverse events were mild to moderate and transient. Grade 3/4 neutropenia, leucopenia, and neutropenic fever were observed in 14 %, 9 %, and 1 % of the cycles, respectively. Twelve percent of patients experienced grade 2/3 mucositis, but only 7 % experienced grade 2/3 hand-foot skin reaction by cycles (Table 6). Although the study design included measuring left ventricular ejection fraction (LVEF) before and after treatment using echocardiography, these measurements were available in only 40 patients. The median LVEF at the end of treatment was not significantly different from that at baseline, even in those patients previously exposed to anthracycline (Table 7).

**Table 6:** Specific toxicities, evaluated by cycles (total 284 cycles)

Toxicity	Grade 1		Grade 2		Grade 3		Grade 4		Total	
	n	%	n	%	n	%	n	%	n	%
Leukopenia	7	(2 %)	18	(6 %)	19	(7 %)	5	(2 %)	49	(17 %)
Neutropenia	4	(1 %)	10	(4 %)	27	(10 %)	12	(4 %)	53	(19 %)
Neutropenic fever	1	(0 %)	2	(1 %)	2	(1 %)	0	(0 %)	5	(2 %)
Anemia	26	(9 %)	25	(9 %)	6	(2 %)	3	(1 %)	60	(21 %)
Thrombocytopenia	16	(6 %)	4	(1 %)	3	(1 %)	0	(0 %)	23	(8 %)
Mucositis	11	(4 %)	27	(10 %)	5	(2 %)	0	(0 %)	43	(15 %)
Hand-foot syndrome	39	(14 %)	16	(6 %)	4	(1 %)	0	(0 %)	59	(21 %)
Nausea	22	(8 %)	5	(2 %)	2	(1 %)	0	(0 %)	29	(10 %)
Vomiting	12	(4 %)	9	(3 %)	6	(2 %)	0	(0 %)	27	(10 %)
Anorexia	38	(13 %)	6	(2 %)	4	(1 %)	0	(0 %)	48	(17 %)
Diarrhea	16	(6 %)	6	(2 %)	0	(0 %)	0	(0 %)	22	(8 %)
Alopecia	35	(12 %)	3	(1 %)	0	(0 %)	0	(0 %)	38	(13 %)

**Table 7:** Change of left ventricular ejection fraction before and after treatment

Population	n	Baseline	After treatment	P valve
Evaluable cases	40	70.93 %	68.59 %	0.115
Patients with Cardiovascular history	18^a^	69.89 %	66.36 %	0.2308^b^
Previous exposure to anthracyclines				
Yes	7	71.14 %	67.57 %	0.85
No	36	70.61 %	68.93 %	0.27

^a^Exclude two subjects only have baseline record

^b^Wilcoxon test

## Discussion

Recent improvements in screening and adjuvant therapies are responsible for the nearly 90 % 5-year survival rate for all breast cancer patients [10]. Nonetheless, except for some cases of oligometastasis, MBC remains an incurable disease with a median survival of less than 2 years. As the first-line therapy, taxane-based regimens provide better response rates (RRs) and longer PFS than anthracycline-based combinations, with a median OS of 19.3 months [11]. However, resistance to these drugs is common and once resistance develops, there is no standard palliative treatment.

As it is common practice to combine anthracycline, taxane, and targeted therapy for neoadjuvant or adjuvant treatments, alternative therapeutic options after recurrence are limited. Different drugs such as capecitabine, vinorelbine, gemcitabine, ixabepilone, and eribulin, either alone or in combination, have been reported to provide therapeutic benefit, including increased RR, PFS, and OS [12–19].

Although all these drugs can be effective when administered to taxane-pretreated patients, additional drug combinations are usually accompanied by increasing adverse effects such as neutropenia, peripheral neuropathy, and mucositis. Long-term adverse effects from previous treatments such as neuropathy from taxane, cardiomyopathy from anthracyclines, and pulmonary fibrosis from radiation may prevent further treatment with the above agents.

PLD is formulated with a polyethylene glycol coating that covers a liposome bilayer containing an aqueous doxorubicin core. Concentrations in tumor tissue can be several-fold higher than those in the adjacent normal tissue [20]. PLD doses are effective in both elderly women with locally advanced or MBC [21] and in patients with advanced breast cancer, even those who have been heavily pretreated. Flegi et al. reported a retrospective study of single-agent PLD in the treatment of MBC. Treatment resulted in an ORR of 26 %, a PFS of 5.8 months, and an OS of 14.2 months [22]. A recently published randomized phase 3 study comparing PLD with capecitabine as the first-line chemotherapy in elderly patients with MBC reported a median PFS of 5.6 *versus* 7.7 months (*P* = 0.11), and a median OS of 13.8 and 16.8 months (*P* = 0.59) for PLD and capecitabine, respectively. Both treatments demonstrated comparable efficacy and acceptable tolerance as first-line single-agent chemotherapies in elderly patients with MBC [23]. In summary, evidence suggests that regimens including PLD as part of a combined therapy are efficacious and safe as a first-line treatment for MBC.

In the present study, all patients had previously received taxane for MBC, while only seven patients had previously received adjuvant anthracycline, and all other patients were naïve to anthracycline, cyclophosphamide, and 5-FU. In the majority of cases, hematologic toxicity was managed by dose reduction and symptomatic treatment with hematopoietic growth factor. The most common non-hematologic toxicities were hand-foot skin reactions (all grades, 21 %; grade 3/4, 1 %), while other adverse effects were mild and manageable. The incidence of severe toxicity was low and resulted in only two patients dropping out of the study. The mean number of treatment cycles received was 5.7 and 5.9 for patients in the ITT and evaluable populations, respectively. The efficiency evaluation was almost the same for these two groups; the ORR was more than 40 % in both populations, and the DCR was more than 80 % in both the groups. Similarly, the median PFS and OS were identical (8.2 months and 36.6 months, respectively).

PLD is suspected to have the advantage of low cardiac toxicity. After following 141 patients, Gill et al. reported that only one patient had a clinically significant decrease in LVEF at a cumulative dose of 1670 mg/m^2^, suggesting that this routine surveillance of LVEF may not be necessary in the absence of other risk factors [24]. Similarly, the current study found that there was no significant decline in LVEF after treatment, including patients who had a history of cardiovascular disease or who were treated with anthracycline prior to the study. To evaluate the effect of PLD as adjuvant chemotherapy, Rayson et al. compared the concurrent administration of trastuzumab and PLD with the sequential administration of anthracycline and trastuzumab as adjuvant chemotherapy. Of the 179 randomized patients, the incidence of cardiac toxicity was 18.6 % in the anthracycline group, compared to 4.2 % in the PLD group [25].

The major weak point of our study was the small sample size and inadequate information on Her2 status, which prevented us from performing further efficiency analyses in the different subgroups. As there is no reported aggravated cardiac toxicity associated with PLD, adding PLD to Her2-targeting therapy is an attractive option. The GEICAM/2004-05 study combined PLD with cyclophosphamide and trastuzumab as the first-line therapy for Her2-positive MBC patients. Among the 48 evaluable patients, the ORR was 68.8 %, the median time-to-progression (TTP) was 12 months (95 % CI: 9–15.1 months), and the median OS was 34.2 months (95 % CI: 27.2–41.2 months). There were no reports of symptomatic heart failure [26].

Several different combinations of PLD have also been reported, including PLD and gemcitabine, which resulted in an ORR of 50 %, and a median PFS and OS of 8.8 months and 19 months, respectively. However, with this combination, seventy-five percent of the patients experienced grade 3 or 4 treatment-related toxicity [27]. PLD in combination with docetaxel was evaluated in two separated studies, and an ORR of 35 %, a median TTP of 9.8 months, and a median OS of 20.6 months were observed, but the incidence of grade 3 and 4 neutropenia was higher than 50 % in each study [28, 29]. Finally, PLD combined with oral vinorelbine results in an ORR of 52 %, and a median PFS and OS of 8.8 months and 24.8 months, respectively. However, symptomatic grade 3 cardiotoxicity and febrile neutropenia occurred in 15 % and 47 % of the patients, respectively [30, 31]. In summary, PLD used as combination therapy results in different treatment efficacies and produces different adverse effects, depending on the drug with which it is combined. Compared to these studies, our study had the lowest toxicities, especially hematologic toxicity, but the determined efficacy was the same.

## Conclusions

In conclusion, the regimen of PLD, cyclophosphamide, and 5-FU combination was associated with promising ORR and PFS, a safe cardiac toxicity profile, and manageable adverse effects. This regimen could be considered as a treatment option for patients with progressed MBC who have undergone taxane-based treatment.
